# Hypervolemia-Induced Immune Disturbances Do Not Involve IL-1ß but IL-6 and IL-10 Activation in Haemodialysis Patients

**DOI:** 10.3390/toxins12030159

**Published:** 2020-03-03

**Authors:** Christof Ulrich, Annegret Wilke, Nadja Schleicher, Matthias Girndt, Roman Fiedler

**Affiliations:** Department of Internal Medicine II, Martin Luther University Halle-Wittenberg, 06120 Halle, Germany; annegret.wilke@uk-halle.de (A.W.); schnaddlinchen@web.de (N.S.); matthias.girndt@uk-halle.de (M.G.); roman.fiedler@uk-halle.de (R.F.)

**Keywords:** haemodialysis, hypervolemia, monocyte, inflammation, bioimpedance

## Abstract

Dysregulated fluid homeostasis is frequent in haemodialysis (HD) patients and is linked to inflammation which may be elicited by endotoxemia. The impact of hypervolemia on immune cells has not been studied in detail. Therefore, we analysed the hypervolemic activation of peripheral blood mononuclear cells (PBMCs) in HD with special focus on the NLRP3 inflammasome response. First, 45 HD were included in the observational study. Immune parameters including cell counts, caspase-1, oxidative stress, cytokine gene expression and serum analysis (IL-1ß, IL-6, IL-10) were all measured at two time points. Fluid status was evaluated by electrical bioimpedance vector analysis, defining hypervolemia (H) as >75 vector percentile. Then, 17 patients were classified as hypervolemic (H-HD), 19 as normovolemic (N-HD) and 9 failed to meet the inclusion criteria. Monocytes were elevated and lymphocytes were decreased by hypervolemia. NLRP3 inflammasome components, caspase-1 and IL-1ß expression were not statistically different between the two groups. Serum IL-6 levels were significantly elevated in H-HD. IL-10 mRNA transcripts were elevated by 2-fold in H-HD but were not efficiently translated. We conclude that the NLRP3 inflammasome is not activated by hypervolemia thus refuting the thesis that endotoxemia may be a main driver for inflammation in H-HD. Nevertheless, inflammation is generally higher in H-HD compared to N-HD patients and is not sufficiently balanced by anti-inflammatory mechanisms.

## 1. Introduction

Extracellular fluid overload (hypervolemia) is highly prevalent in end-stage renal failure patients and is a powerful predictor of mortality [1,2]. Among many causes, inflammation, hypoalbuminemia and increased capillary permeability contribute to an altered fluid distribution between blood volume and the interstitial fluid compartments. Bioimpedance analysis (BIA) provides a non-invasive technique to monitor fluid dynamics in end-stage renal disease (ESRD) [3,4,5] and is a valuable means for achieving normovolemia in these patients. 

The disturbed salt and water homeostasis from which chronic kidney disease patients suffer can lead to oedema, dysregulated blood pressure and congestive heart failure [6,7]. However, abnormalities in the fluid state also appear to be linked to inflammation. Elevated VCAM-1 and IL-6 levels were found in overhydrated haemodialysis (HD) patients [8]. The exact pathomechanism is not known, but translocation of endotoxins through the oedematous bowel walls is considered as a possible source of inflammation in haemodialysis patients with fluid excess. 

Proinflammatory monocytes are elevated in dialysis patients with a high atherosclerosis risk profile [9,10]. They contribute to inflammation via a secretion of inflammatory and anti-inflammatory cytokines [11]. To the authors’ knowledge, the molecular effects of hypervolemia on immune cells have not been investigated in depth. Monocytes are part of the regulatory network combining tasks in innate and adaptive immunity. Bacterial products, cytokines and cell stress induce the monocytic NLRP3 inflammasome machinery. The canonical NLRP3 activation concept is a two-signal model. The first signal, which is provided by microbial components or endogenous cytokines, primes the NLRP3 inflammasome by up-regulating NLRP3, pro-IL-1ß and pro-IL-18. The second signal, which is triggered by diverse mechanisms (e.g., ionic K+ gradient, ATP, nigericin, reactive oxygen species (ROS), uric acid crystals), is the caspase-1-dependent cleavage of the pro-enzymes [12]. However, recently, Gaidt et al. raised an alternative inflammasome activation concept for primary monocytes, demonstrating that lipopolysaccharide (LPS) alone is sufficient for IL-1ß processing. This non-canonical model is independent of pyroptosis—a special form of cell death [13]. Following Gaidt et al.’s model, endotoxemia-laden HD patients should show a concise NLRP3 activation.

Currently, it is unclear whether hypervolemia produces inflammation or inflammation contributes to hypervolemia. Notwithstanding, both are known to contribute to the aggravation of the morbidity and the mortality risk of HD patients [14,15].

Therefore, we investigated in this cross-sectional observational study the impact of uremic hypervolemia on cytokine activation, with a special focus on NLRP3-dependent IL-1ß activation.

## 2. Results

According to the bioimpedance analysis, 17 HD patients were classified as H-HD and 19 were N-HD. The phase angle used as an indicator of membrane integrity and water distribution between intra- and extracellular spaces was 4.6 ± 0.8° for H-HD and 6.0 ± 0.7° for N-HD patients (*p* < 0.001). 

### 2.1. Hypervolemia: Weight Gain and Clinical Signs of Inflammation

Hypervolemia is associated with weight gain and higher C-reactive protein (CRP) levels (Table 1). The residual urine volume of H-HD and N-HD patients was not statistically different (Table 1). We found that 8 H-HD and 4 N-HD patients were without any residual urine excretion (*p* = 0.158). The ultrafiltration volumes (l) were statistically different (H: 3.4 ± 0.6 vs. 2.7 ± 0.6, *p* = 0.021) and the intradialytic change in blood volume was −5.4% ± 4.7 and −9.5% ± 4.9, respectively (H vs. N, *p* = 0.016). These changes, however, had no significant effect on the mean arterial blood pressure (MAP). Kt/V, the indicator of dialysis efficacy, remained severe in overhydrated patients (Kt/V (1) vs. Kt/V (2)) as measured after the long dialysis-free interval, whereas in normovolemia there was some normalisation of dialysis quality. The Charlson comorbidity index (CCI) was not statistically different for both groups (Table 1). 

### 2.2. Hypervolemia is Associated with Higher Monocyte and Lower Lymphocyte Levels

**Figure 1:**
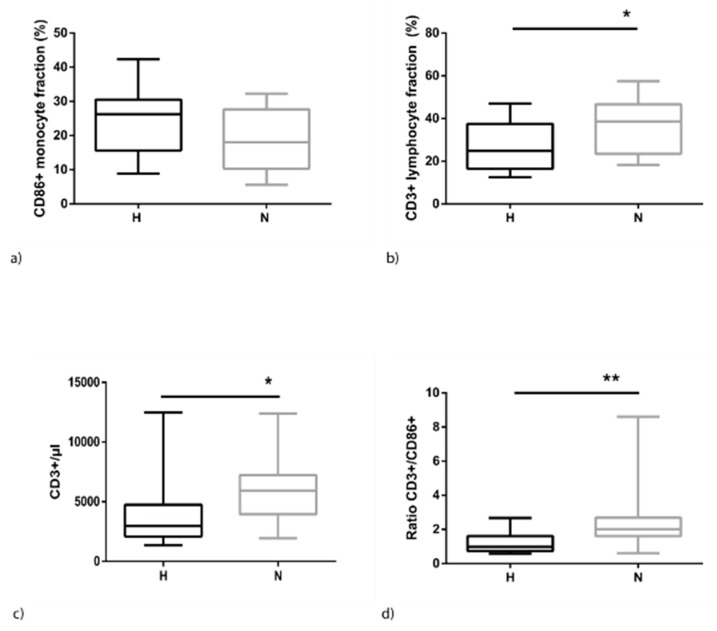
Distribution of monocytes and lymphocytes among peripheral blood mononuclear cells (PBMCs) of hyper- (H) and normovolemic (N) haemodialysis (HD) patients at BS2. (**a**) Percentage of monocytes staining positive for CD86; (**b**) percentage of lymphocytes staining positive for CD3; (**c**) absolute numbers of lymphocytes; and (**d**) ratio of lymphocytes to monocytes. The data are presented as box blots depicting median as well as the 25th and 75th percentiles. Statistical analysis was performed using the Mann–Whitney test for unpaired samples (* *p* < 0.05; ** *p* < 0.01).

Fluid overload in HD patients is associated with an increase of CD86-positive monocytes. This holds true for relative (Figure 1) as well as absolute values (H: 1110.4 ± 891.3 vs. N: 764.7 ± 541.8, *p* = 0.191). In contrast, CD3+T-cells are significantly reduced (Figure 1). This results in a skewed distribution of the proportion of both cell types. Optimal ratios for immune cell interactions range from 1:3 to 1:7 (monocyte: T-cell) but, in hypervolemia, this ratio is shifted towards lower levels of around 1:8 directly after dialysis (blood sampling 1, BS1) and decreasing to 1:2 at BS2 (H: 1.2 ± 0.6 vs. N: 2.5 ± 1.8, *p* < 0.008).

### 2.3. Hypervolemia: Low Impact on the NLRP3 Inflammasome

**Figure 2:**
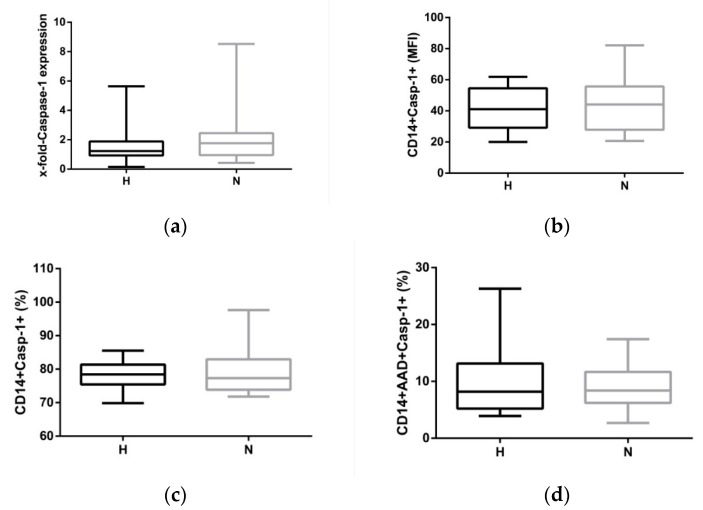
Caspase-1 expression of activated CD14+ monocytes at BS2. (**a**) mRNA expression of caspase-1 in hypervolemic (H) and normovolemic (N) patients; (**b**) protein expression (median fluorescence intensity (MFI)) in CD14+ monocytes staining positive for caspase-1; (**c**) frequency of monocytes staining positive for caspase-1; (**d**) AAD and caspase-1 double-positive monocytes representing the range of pyroptosis in both cohorts. The data are presented as box blots depicting median as well as the 25th and 75th percentiles. Statistical analysis was performed using the Mann–Whitney test for unpaired samples.

The variety of stimuli activating the NLRP3 inflammasome led to speculation that volume overload may also activate the central inflammatory pathway in monocytes. Firstly, oxidative stress was analysed to see if it was present in both groups to different extents. As mitochondria are thought to be a major source of ROS, mitochondrial ROS formation was analysed. Neither the frequency of monocytes staining positive for the ROS indicator (H: 62.9 ± 13.1% vs. N: 54.7% ± 16.3, *p* = 0.122) nor the expression density (median fluorescence intensity (MFI)) of ROS-positive cells were different between the two groups (H: 8.8 ± 1.2 vs. N: 7.0 ± 0.8, *p* = 0.196). In line with this strategy, caspase-1 expression was analysed. mRNA expression of pro-caspase-1 is a prerequisite for the clustering of the activated NLRP3 platform which finally cleaves pro-IL-1ß into bioactive IL-1ß. Caspase-1 mRNA expression was not statistically different between H-HD and N-HD patients, neither directly after dialysis (BS1; Table 2) nor after the long dialysis-free interval (Figure 2a). However, there was a distinct difference in the frequency of cells staining positive for caspase-1 and 7-aminoactinomycin D (7-AAD) between H-HD and N-HD patients. It is speculated that this difference was due to a slightly higher pyroptotic death rate in H-HD patients (Table 2), but this difference was not observed at BS2 (H: 9.8 ± 1.4% vs. N: 8.9 ± 0.9%, *p* = 561).

Caspase-1 activity data (Figure 2b,c) and mRNA results (Figure 2a)—there was no sufficient evidence to suggest that the monocytic NLRP3 inflammasome is activated differently in fluid-overloaded patients. Even pyroptosis, a form of cell death, as measured by AAD and caspase-1 double-positive staining did not differ in both cohorts (Figure 2d).

**Figure 3:**
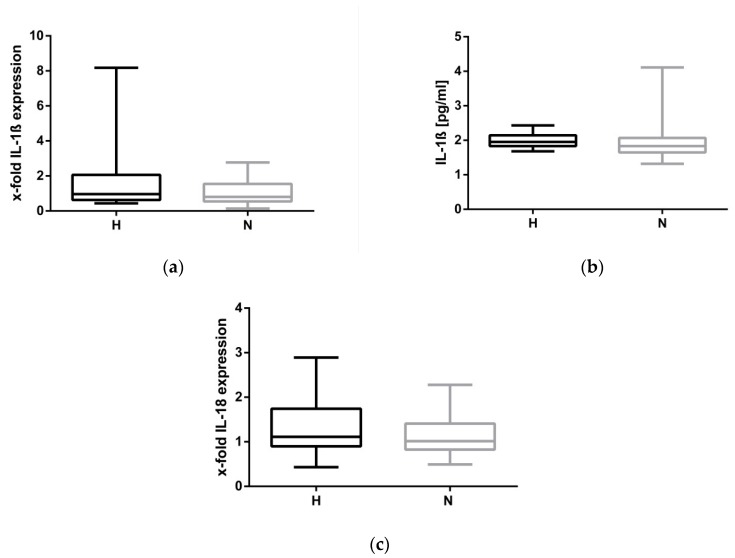
IL-1ß and IL-18 expression at BS2. (**a**): mRNA expression of IL-1ß in hypervolemic (H) and normovolemic (N) patients; (**b**): Serum IL-1ß protein expression (pg/mL); (**c**) mRNA expression of IL-18. The data are presented as box blots depicting median as well as the 25th and 75th percentiles. Statistical analysis was performed using the Mann–Whitney test for unpaired samples.

To finally establish that the NLRP3 inflammasome was not activated in H-HD patients, IL-1ß RNA and serum protein levels were measured. As demonstrated in Figure 3a,b, fluid overload appears to not be associated with IL-1ß expression and secretion. The impression that the NLRP3 inflammasome is not activated by hypervolemia is strengthened by data showing that the mRNA level of IL-18, a cytokine which is also regulated by the NLRP3 complex, is not different in H-HD and N-HD patients (H: 1.3 ± 0.6 vs. H: 1.1 ± 0.4, *p* = 0.222, Figure 3c).

The initial speculation that inflammation in dialysis patients was induced either by bacterial products or by cytokines provides the NLRP3 inflammasome priming signal. Hypervolemia and its following consequences, respectively, provide the second signal for inflammasome activation and had to be verified by ex vivo stimulation of peripheral blood mononuclear cells (PBMCs) with LPS and nigericin. LPS/nigericin stimulation significantly activated caspase-1-associated pyroptosis (%Casp-1+AAD+) compared to unstimulated samples (*p* < 0.001); however, there was no evidence to suggest that hypervolemia extended the stimulatory power of the artificial stimuli (Table 3).

### 2.4. Compensatory Mechanisms Evoked by Hypervolemia

**Figure 4:**
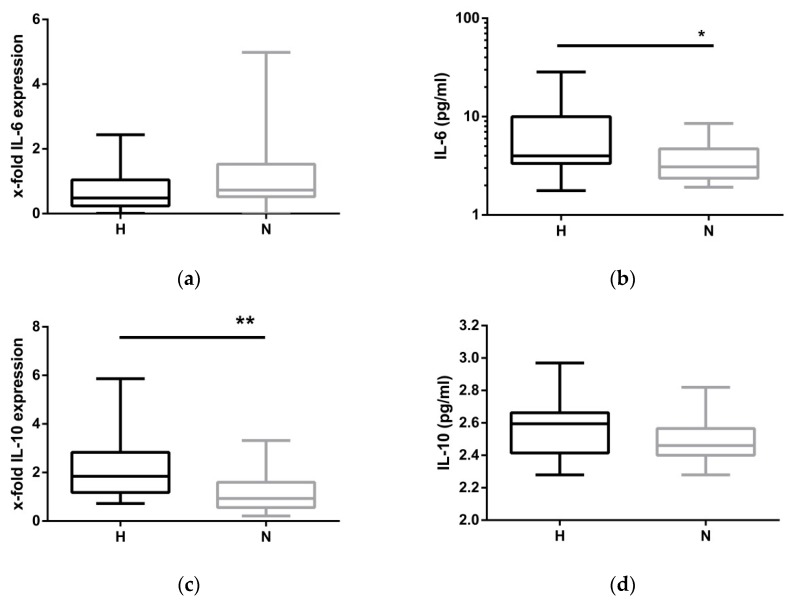
IL-6 and IL-10 expression at BS2. (**a**,**c**) mRNA expression of IL-6 and IL-10 in hypervolemic (H) and normovolemic (N) patients; (**b**,**d**) Il-6 and IL-10 expression (pg/mL) in serum of hyper- and normovolemic patients. The data are presented as box blots depicting median as well as the 25th and 75th percentiles. Statistical analysis was performed using the Student’s *t*-test or the Mann–Whitney test for unpaired samples as appropriate. (* *p* < 0.05, ** *p* < 0.01).

Hypervolemia is speculated to trigger inflammation. While mRNA expression of IL-1ß remained unchanged between H-HD and N-HD patients, the observation was extended by measuring IL-6 (Figure 4a,b), a potent inflammatory stimulus and IL-10, an important anti-inflammatory cytokine (Figure 4c,d). At the protein level, the proinflammatory cytokine IL-6 was significantly elevated in hypervolemia (Figure 4b). To balance inflammation, monocytes and different subsets of lymphocytes produce the anti-inflammatory cytokine IL-10. A 2-fold increase in IL-10 mRNA expression was found in fluid-overloaded patients (Figure 4c). However, the amount of serum IL-10 protein did not correspond with the amount of IL-10 mRNA (Figure 4d).

## 3. Discussion

Chronic kidney failure often leads to an accumulation of excess water which compromises the health of patients in many ways. One aspect of concern is the role of fluid overload on blood pressure. Extracellular fluid expansion increases the plasma volume and this may directly affect blood pressure. In our small study, blood pressure measured at two different time points did not significantly differ between normo- and H-HD patients. Therefore, we support data from Antlanger et al., who saw no relationship between the fluid status and blood pressure [16]. In contrast to this, other studies report a significant positive correlation between fluid overload and blood pressure [6,17]. However, it must be noted that blood pressure varies widely and other factors such as the sympathetic nervous system and the renin–angiotensin system, may contribute to arterial hypertension to a higher extent.

Regarding the inflammatory aspects induced by hypervolemia, there remains in a way the classical “chicken and egg” dilemma. It is not understood whether inflammation or hypervolemia develops first. There are various studies showing associations between fluid overload and systemic inflammation [6,18], but more convincing evidence that hypervolemia is the catalyst of inflammation in dialysis patients is provided by Gonçales et al., who reported a relationship between fluid overload and endotoxemia [19]. In line with these data, McIntyre et al. published a study showing that the amount of endotoxin was significantly increased by dialysis-induced stress, in terms of high ultrafiltration rates leading to subclinical gut ischemia and translocation of endotoxin from the gut [20]. Noting that bacterial products can stimulate the alternative NLRP3 pathway [21], it was hypothesized that caspase-1 activity as well as IL-1ß expression would increase in fluid-overloaded haemodialysis patients. However, any activation of the NLRP3 complex was not demonstrated in our study. Although some oxidative stress could be shown in PBMCs, potentially providing the input for “the second signal” or enhancing this kind of signalling, our in vitro stimulation (LPS/nigericin) data support the conclusion that the NLRP3 inflammasome is not regulated differently in H-HD and N-ND patients. Nevertheless, the inflammatory cycle present in HD exists at a higher extent in H-HD patients. In agreement with Mitsides et al., elevated IL-6 protein levels were observed in H-HD compared to N-HD [8]. This observation was extended by demonstrating that as a matter of principle, hypervolemic signalling evoked an anti-inflammatory response, as seen by significantly increased IL-10 mRNA levels in H-HD. Monocytes and special lymphocyte subsets (Th2, Tr1, Th17, Th1, B cells) are the main sources of IL-10. Impairment of these cells to translate IL-10 mRNA efficiently may be one reason why IL-10 protein levels do not significantly differ between H-HD and N-HD. The regulation of IL-10 is complex and there is evidence that the associated anti-inflammatory gene is post-transcriptionally and post-translationally regulated by miRNA [22,23]. Decreased lymphocyte cell counts must also be considered in hypervolemia as these may hinder optimal T-cell responses. All in all, these observations remind one of the uremic immune defect ending in a compromised immune system, for example, in non-responsiveness to the hepatitis B vaccination [24,25] or, generally speaking, in an insufficient feedback inhibition of proinflammatory cytokines [26,27]. The inhibition of immune cells to mount an appropriate anti-inflammatory response remains a challenging task to unravel in overhydrated haemodialysis patients.

We acknowledge the following limitations of this study. The study was very small but it was designed as a pilot study to get an insight to the possible impact of fluid overload on immunoregulatory cells. Although the study clearly shows that hypervolemia does not activate the NLRP3 inflammasome, observational data have been added with regards to the disturbed anti-inflammatory response in H-HD patients. Furthermore, it must be noted that the endotoxin levels in the patients were not measured. The correct determination of endotoxin in H and N patients would support the interpretation of our data. It must be noted that the association between endotoxemia with unexplained inflammation is controversially discussed and an accurate assay for the detection of endotoxin in the blood is still required (reviewed in [28]). On the other hand, the conclusiveness of such a determination would be difficult. Furthermore, a healthy control group was not incorporated into this study. This would have been advantageous to gain insight into the differences of inflammasomal activation between HD patients and healthy subjects.

## 4. Conclusions

The lacking IL-ß response in our overhydrated haemodialysis patients challenges the data of colleagues showing that inflammation is triggered by microbial products, i.e., by endotoxemia. It is clear that H-HD patients are confronted with a higher burden of inflammation compared to N-HD patients. One main problem is finding an adequate anti-inflammatory balance for these patients. In our opinion, overhydration results in an additional inflammatory stimulus aggravating an existing uremic inflammation loop.

## 5. Materials and Methods

### 5.1. Study Population

From 88 dialysis patients of the outpatient dialysis centre of the Department of Internal Medicine II of the University Halle-Wittenberg, Halle, Germany, 43 patients had to be excluded from the study because of a satisfied renal rest function with general low ultrafiltration rates (*N* = 18), lack of willingness (*N* = 7), acute or chronic infections (*N* = 6), peritoneal dialysis (N = 6), corticosteroid/immunosuppressive therapy (*N* = 5), or active tumour disease (*N* = 1). Furthermore, patients under the study procedure who failed to reach a minimum ultrafiltration volume of 2 litres were also excluded (N = 9) from the study. Therefore, 36 patients were enrolled. The reasons for kidney failure were glomerulonephritis (33.3%), diabetic nephropathy (22.2%), interstitial nephritis (11.1%), vascular nephropathy (13.9%) and others (19.4%). Fluid status and bioelectrical impedance vector analysis (Piccoli et al., [4]) were supervised by Haemo-Master (DBB-EXA, Nikkiso Europe GmbH, Langenhagen, Germany). Normohydration was defined as a range of impedance vectors falling within the reference 75% tolerance interval. Overhydration (hypervolemia) was defined by the Piccoli vector diagram >75th percentile. After the dialysis session, the hydration status of the patients was determined. The patients were allocated to the corresponding groups (H, N) and blood samples were drawn (blood sampling (1), BS1). The patients were regularly dialysed thrice weekly, every other day. As the biological consequences of fluid overload may aggravate over the weekend (long dialysis-free interval), blood sampling was repeated (2, BS2).

CRP, albumin, creatinine, and haemoglobin were measured monthly in a certified clinical laboratory using standardised methods. Dialysis quality determination is an integrated feature of the Nikkisio-DBB-EXA dialysis monitor capable of measuring the dialysis quality (Kt/V) by the single-pool method. The study was conducted according to the Declaration of Helsinki. Written informed consent was obtained from all study subjects and the study protocol was approved by the local ethics committee.

### 5.2. PBMC isolation

Peripheral blood mononuclear cells (PBMCs) were isolated from blood samples anti-coagulated with EDTA by ficollisation (GE Healthcare, Solingen, Germany). The quality of isolated cells was tested by 7-AAD (ThermoFisher Scientific, Darmstadt, Germany) staining. The vitality of PBMC was 99.5 ± 1.0.

### 5.3. NLRP3 Inflammasome Stimulation Model

Initially, 0.25 Mio cells suspended in RPMI/2%FCS/1%glutamine were incubated at 37 °C at 5% CO_2_ atmosphere for 2 h. Lipopolysaccharide (LPS, 1 µg/mL, Sigma-Aldrich, Steinheim; Merck-Millipore, Darmstadt) and nigericin (5 µg/mL, Sigma-Aldrich) were applied as stimuli. In contrast to LPS, nigericin was applied for the last 15 min of the incubation period. Negative controls were without stimulus. Basal activation of cells was measured directly without cultivation.

### 5.4. Antibodies for Flow Cytometry

The following antibodies were used: anti-CD16 (clone 3G8, ThermoFisher Scientific), -CD86 (clone HA5.2B7, Beckman Coulter, Krefeld, Germany), -CD14 (clone 61D3, ThermoFisher Scientific and clone TÜK4, Miltenyi Biotec), -CD15eF450 (clone HI98, ThermoFisher Scientific), -CD3 (clone BW264/56, Miltenyi Biotec, Bergisch-Gladbach, Germany), 7-AAD (ThermoFisher Scientific). Absolute cell counts were enumerated using CountBright™ absolute counting beads (ThermoFisher Scientific). Samples were analysed on the MACS Quant analyser (Miltenyi Biotec) using MACS Quantify software. Gates were set according to fluorescence minus One FMO controls.

### 5.5. Caspase-1-Assay

Caspase-1 was detected by flow cytometry with the FAM-FLICA^®^ Caspase Assay using the FAM-YVAD-FLICA inhibitor probe as described by the manufacturer (BioRad, Feldkirchen, Germany). The fluorescent inhibitor binds to activated caspase-1. One h before ending the regular incubation period, cells (0.25 Mio) were pelleted and resuspended in sterile PBS/0.5% HSA containing the casp-1 inhibitor probe. The incubation was continued for 1 h. Samples without caspase-1 inhibitors were used as negative controls. Cells were counterstained with anti-monocyte and lymphocyte-specific antibodies. 7-AAD (ThermoFisher Scientific) staining in combination with caspase-1 positivity was applied for the detection of pyroptosis (AAD+Casp-1+).

### 5.6. Mitochondrial Oxidative Stress

Mitochondrial stress was determined by flow cytometry using the fluorgenic indicator probe MitoSOX™ (ThermoFisher): 250,000 PBMCs were cultivated under unstimulated and stimulated conditions for 2 h. For the last 15 min of the incubation period, cells were labelled with anti-CD3, -CD14 and MitoSOX™ (2 µM). Cells without MitoSOX™ staining served as negative controls.

### 5.7. RNA/cDNA/qPCR

For the detection of caspase-1 and cytokine transcripts, whole blood was drawn in TEMPUS^™^ Blood RNA Tubes (Life Technologies). Samples were stored at −80 °C as recommended and an analysis was done after collection of all blood samples.

RNA was isolated using the Tempus^TM^ Spin RNA Isolation Reagent Kit (Life Technologies, Darmstadt, Germany). The RNA concentration and quality (260/280 ratio: 2.05 ± 0.005) were tested by NanoDrop technique (PEQLAB Biotechnologie GmbH, Erlangen, Germany). Equal amounts of RNA (500 ng) were reverse transcribed using the FastGene Scriptase Basic cDNA Kit (Nippon Genetics Europe, Düren, Germany).

Caspase-1 (Hs00354836_m1), IL-1ß (Hs00174097_m1), IL-6 (Hs99999903_m1), IL-10 (Hs00961622_m1) and ACTB (Hs01060665_m1)) mRNA expression were analysed using TaqMan probes (Life Technologies) using qPCRBIO Probe Mix High-ROX (Nippon). The samples were processed in duplicates on a StepOnePlus Cycler (Life Technologies). Data were normalised by actin B and related to healthy control donor RNA. Thus, results are expressed as x-fold difference (RQ) compared to the healthy control.

### 5.8. Cytokine Analysis

IL-6, IL-10 and IL-1ß serum levels were analysed using a BD™ cytometric Bead Array (CBA, BD Biosciences, Heidelberg, Germany). The enhanced sensitivity flex sets can detect as low as 0.274 pg/mL of each cytokine.

### 5.9. Data Analysis

Results are expressed as mean ± SD unless otherwise indicated. Categorical variables were statistically analysed by Fisher’s exact test. All continuous variables were controlled for normal distribution using the D’Agostino–Pearson omnibus test. Continuous data were compared using the Student’s *t*-test or Mann–Whitney test for unpaired samples. The initial statistical power calculation was done by G*Power (http://psycho.uni-duesseldorf.de/abteilungen/aap/gpower3). To get a statistically significant difference in the expression of caspase-1 per monocyte (MFI) between both groups, 15 patients of each group should be included (*t*-test, difference between two independent means, actual power of 0.95, the α-error probability 0.05, effect size d = 1.32).

All calculations were carried out using the SPSS 21.0 (SPSS Inc., Chicago, IL, USA) or GraphPad Prism 6.0 statistics software (GraphPad Software Inc., La Jolla, CA, USA). The level of significance was set at *p* < 0.05.

## Figures and Tables

**Table 1:** Demographic data of hyper- (H) and normovolemic (N) patients.

Patient’s Characteristics	H (*n* = 17)	N (*n* = 19)	Statistics
Age (years)	62.2 ± 17.1	56.2 ± 15.8	0.286
Sex (f, %)	35.3	26.3	0.721
Diabetes (%)	47.1	15.8	0.070
BMI (Kg/m^2^)	29.7 ± 6.7	26.2 ± 4.9	0.090
Dialysis vintage (years)	3.3 ± 3.0	5.1 ± 5.2	0.235
Dialysis (hours/week)	13.4 ± 1.3	12.7 ± 1.4	0.115
Kt/V (BS1)	1.3 ± 0.3	1.3 ± 0.2	0.267
Kt/V (BS2)	1.2 ± 0.2	1.4 ± 0.3	0.051
Residual urine volume (mL)	541.2 ± 529.5	581.1 ± 468.8	0.158
CRP (mg/dL)	12.9 ± 11.6	7.0 ± 8.9	0.056
Albumin (g/L)	38.5 ± 4.7	40.3 ± 2.5	0.252
Haemoglobin (mmol/L)	6.7 ± 0.8	6.8 ± 0.8	0.643
Creatinine (µmol/L)	778 ± 280	879 ± 320	0.327
ECW (L)	20.8 ± 5.0	15.5 ± 3.1	0.001
ICW (L)	26.4 ± 5.0	24.2 ± 3.5	0.144
TBW (L)	47.2 ± 9.6	39.8 ± 6.4	0.001
ECW/TBW (%)	44.0 ± 3.6	37.2 ± 1.8	0.001
Interdialytic weight gain (kg)	3.4 ± 0.9	2.7 ± 0.6	0.018
Hypertensive drugs (*n*)	2.8 ± 0.4	2.4 ± 0.4	0.535
Diuretic drugs (*n*)	0.8 ± 0.2	1.3 ± 0.2	0.054
MAP (mm Hg, BS1)	102 ± 20	100 ± 15	0.757
MAP (mm Hg, BS2)	108 ± 15	110 ± 21	0.781
**CCI**	6.1 ± 2.2	5.3 ± 2.6	0.323

BS1: blood sampling 1; BS2: blood sampling 2. ECW: extracellular water, ICW: intracellular water, TBW: total body water, MAP: mean arterial pressure. CRP: C-reactive protein, CCI: Charlson comorbidity index.

**Table 2:** Caspase-1-related parameters at BS1. MFI, median fluorescence intensity; 7-AAD, aminoactinomycin D.

Caspase-1 Expression/Frequency	H	N	*p*-Value
Caspase-1 mRNA (x-fold)	1.4 ± 1.3	2.1 ± 2.1	0.643
CD14+Casp-1+ (MFI)	46.0 ± 16.7	43.1 ± 29.9	0.243
CD14+Casp-1+ (%)	77.5 ± 4.7	79.2 ± 6.2	0.370
CD14+Casp-1+AAD+ (%)	10.2 ± 4.7	7.9 ± 3.7	0.046

**Table 3:** Caspase-1-related parameters at BS2 under stimulated conditions.

Caspase-1 Expression/Frequency	H	N	*p*-Value
CD14 + Casp-1 + (MFI)	89.9 ± 22.6	88.0 ± 23.3	0.900
CD14 + Casp-1 + (%)	89.65 ± 4.4	88.5 ± 5.7	0.749
CD14 + Casp-1 + AAD + (%)	54.8 ± 22.1	52.3 ± 20.9	0.720
